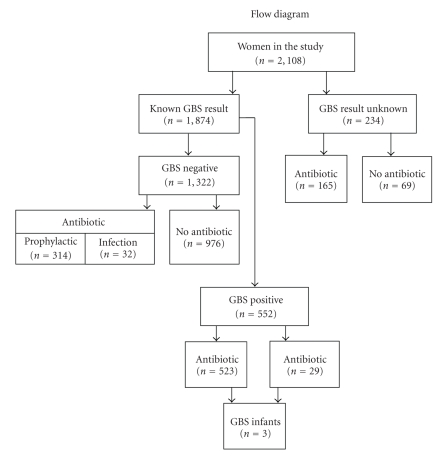# Screening for Group B Streptococcus: A Private Hospital's Experience

**DOI:** 10.1155/2010/451096

**Published:** 2010-06-23

**Authors:** Sebastian Faro, Brenda Brehm, Frances Smith, Melanie Mouzoon, Anthony Greisinger, Oscar Wehmanen, Mark A. Turrentine

**Affiliations:** ^1^The Woman's Hospital of Texas, 7600 Fannin Street, Houston, TX 77054, USA; ^2^Kelsey Research Foundation, 5615 Kirby Drive, Suite 660, Houston, TX 77005, USA; ^3^Department of Obstetrics and Gynecology, Kelsey-Seybold Clinic, 7900 Fannin Street Suite 2100, Houston, TX 77054, USA

## Abstract

*Objective*. To assess the effect of universal screening and administration of intrapartum antibiotic prophylaxis to prevent early-onset neonatal GBS sepsis at a private tertiary care hospital since issuance of the 2002 CDC guidelines for preventing perinatal GBS disease. *Methods*. Retrospective analysis of women delivering between January 1, 2003 and December 31, 2004 at a private tertiary care hospital in Houston, Texas. The percentage of women screened, GBS positive women receiving intrapartum antibiotic prophylaxis, and infants developing early-onset GBS sepsis were determined. *Results*. 2,108 women delivered 2,135 infants with 1,874 (89%) screened for GBS. Of those screened, 1,322 (71%) tested negative and 552 (29%) tested positive for GBS. In this analysis of 2,135 infants, 3 (0.94 cases/1,000 live births) were diagnosed with invasive GBS sepsis. *Conclusion*. High rates of screening of pregnant women for GBS colonization and use of intrapartum antibiotic prophylaxis for GBS carriers can be achieved in a private tertiary care hospital setting. “Synopsis: High screening rates for group B streptococcus in a private tertiary care hospital reduce the incidence of maternal and early onset neonatal GBS infection.”

## 1. Introduction

Streptococcus agalactiae (group B streptococcus (GBS)) is one of the most common bacterial causes of life-threatening infection in newborns [1, 2]. GBS was first reported as a human pathogen in 1938, but it was not until the 1970s that GBS was described as a major pathogen responsible for neonatal sepsis, pneumonia, and meningitis [3]. GBS neonatal infection is divided into two categories: early-onset disease, which occurs within the first week of life, and late-onset infection, which occurs between one week to 3 months of age [4]. GBS vaginal colonization occurs in 4% to 40% of pregnant and nonpregnant women and appears to be dependent upon geographical location [5–9]. The GBS bacterium also may lead to chorioamnionitis, myonecrosis of the uterus, neonatal pneumonia, premature delivery, premature labor, premature rupture of amniotic membranes, postpartum endometritis, and septic abortion [10–14]. Furthermore, both mother and newborn infant may experience bacteremia, which can cause both septic shock and death. 

Prior to extensive prevention efforts in the 1990s, the incidence of invasive neonatal GBS infection ranged from 2 to 3 cases per 1,000 live births [12]. In 1996, the Centers for Disease Control and Prevention (CDC) issued guidelines recommending the use of intrapartum antibiotic prophylaxis and by 1999, the incidence of early-onset GBS infection was reduced to 0.5 cases per 1,000 live births [12]. 

In 2002, in order to further decrease the incidence of GBS sepsis, the CDC issued revised guidelines that recommended universal screening of pregnant women between 35 and 37 weeks of gestation [12]. However, GBS infection continues to be a considerable problem, causing significant morbidity and mortality in mothers and their newborn infants. The CDC reported for the period 2000–2002, the average early-onset disease incidence was 0.49 cases per 1,000 live births [14]. Following the revised CDC recommendations in 2002, average early-onset disease incidence decreased to 0.33 cases per 1,000 live births [14]. 

The purpose of this retrospective study was to determine the screening rate for GBS, the incidence of maternal GBS colonization and early-onset neonatal GBS sepsis. In addition, we determined compliance with the CDC recommendation for the use of intrapartum antibiotic prophylaxis for GBS positive women in a private tertiary care hospital after the 2002 CDC recommendation for universal screening of pregnant women for GBS colonization.

## 2. Materials and Methods

The study was conducted at The Woman's Hospital of Texas (WHT), Kelsey-Seybold Clinic (KSC), and the Kelsey Research Foundation (KRF). The WHT is a private tertiary care hospital with private physicians, a level III nursery that accepts maternal-fetal transfers, and the only specialty hospital in Houston, Texas focused on the care of women and infants. Approximately 22% of all WHT deliveries each year are high risk. KSC is a large, multispecialty medical clinic with 300 physicians that serves an ethnically diverse population of over 400,000 patients at 18 locations in Houston, Texas. The KRF is a 501 (c) (3) nonprofit that collaborates with healthcare and research institutions in the Texas Medical Center to conduct health services research, provide patient education, and develop quality improvement initiatives. 

Since 1995, the KRF has maintained a database of clinical information from both WHT and KSC about the pregnancy experience of over 20,000 women at KSC. The database contains information describing the pregnancy experience and outcomes of mothers and infants who received care at WHT and KSC. Data for all (9) obstetricians at KSC who routinely performed deliveries at WHT are included in this study. 

The database was queried to identify the number of deliveries performed during the two-year period (January 1, 2003 through December 31, 2004). Outcome variables included the number and percentage of pregnant mothers screened for GBS, the GBS status of those women screened, the rate of intrapartum antibiotic usage, and the incidence of early-onset GBS sepsis. Sepsis is defined as a positive blood or spinal culture, or both, in addition to the clinical signs and symptoms of infection. Demographic variables, including age, ethnicity, and insurance status, also were collected from the administrative database. Data was analyzed using Microsoft EXCEL and ACCESS (2003, version 11.6355.6360 SP1) software. Because this is a retrospective analysis, waiver of consent was approved by the Institutional Review Board of WHT.

At 35 to 37 weeks gestation, a standard cotton aerobic bacterial culture swab was gently inserted into the lower third of the vagina and then in a single motion, the perineum was swabbed and the external anal sphincter was swabbed. The swab was then placed in Stuart's transport medium and sent to the laboratory at room temperature. All specimens were sent to a commercial laboratory as directed by patients' insurance providers. A screening culture was completed on arrival in labor and delivery if pregnancies were <35 weeks. If GBS bacteriuria was diagnosed during a pregnancy, the patient was considered colonized and treated during labor as per the CDC guidelines [12]. All women who had GBS positive cultures were given antibiotic prophylaxis in labor as recommended by the CDC guidelines [12]. Mothers allergic to penicillin were treated according to 2002 CDC guidelines [12].

## 3. Results

For the two-year study period, 2,108 women delivered 2,135 infants. Of these 2,108 deliveries, 8% were less than 37 weeks gestation. The mean gestational age at delivery was 38 ± 2.31 weeks. There were 39 sets of twins (1.8%), 20 infant deaths (0.9%), and 171 (8%) premature deliveries. (Prematurity is defined as infants born at a gestational age less than 37 weeks.)

The population was 40% African-American, 25% Caucasian, 28% Hispanic, 5% Asian, and 2% other. Approximately 3% of the women in the sample were less than 18 years of age, 56% were 18–30 years of age, 39% were 31–40 years of age, and 2% were 41 or older. The majority (71%) of women were insured through an HMO, while 27% had coverage with a PPO, 2% with Medicaid, and fewer than 1% were self-insured. The demographics of mothers who were GBS positive, GBS negative or those receiving intrapartum antibiotic prophylaxis were comparable to the demographics of the study sample. Chi-square tests were performed and there were no statistical differences in the subgroups.

Among the 2,108 mothers, 1,874 (89%) were screened for GBS and, of these, 1,322 (71%) tested negative and 552 (29%) tested positive for GBS. There were 231 (11%) of the 2,108 mothers with an unknown GBS status. 

Thirty-two (1.5%) of the 2,135 infants in the sample were evaluated for sepsis during their hospitalizations. The sepsis workup included a CBC, blood culture, and lumbar puncture for spinal fluid analysis and culture. Gestational age for 21 (66%) of these infants with sepsis was <30 weeks, for 8 (25%), gestational age was 30 to 36.7 weeks, and for 3 (9%), gestational age was ≥37 weeks. These infants were cultured for sepsis at birth and received antibiotics until results were available. For the majority (30) of these infants, culture results were negative and antibiotics were discontinued. For the two culture positive infants, one had positive blood cultures for GBS and the other had positive blood cultures for coagulase negative staphylococci, and none had meningitis. Thirty-one of the 32 (97%) infants were admitted to the NICU and had admissions of more than 30 days. Of these infants, one developed GBS sepsis. 

Of the 1,322 mothers who tested negative for GBS, 346 (26%) received antibiotic prophylaxis. Of the 346 that received antibiotics, 314 (91%) received antibiotics for surgical prophylaxis (C-section) and 32 (9%) received antibiotics for acute infection (chorioamnionitis, pyelonephritis, and respiratory infection). Among the 314 women who received antibiotics for surgical prophylaxis, 43 (14%) delivered vaginally. Interestingly, these 43 women, although initially GBS negative by culture at 35–37 weeks, met the CDC risk-based criteria for GBS prophylaxis when admitted to labor and delivery. 

A total of 523 (95%) of the 552 mothers who were GBS positive received intrapartum antibiotic prophylaxis. The three GBS positive infants were born to GBS positive mothers and all three had a gestational age <36 weeks (33.5, 32.5, and 25.2 weeks). The mother who delivered her baby at 33.5 weeks had severe chorioamnionitis with artificial rupture of membranes and delivered vaginally on the day she was admitted. She received prophylactic ampicillin and subsequently tested positive for GBS. The second mother delivered twins at 32.5 weeks by repeat C-section on the day she was admitted. The mother had premature rupture of membranes with GBS status unknown at time of delivery and subsequently tested positive for GBS. One twin was GBS positive and had intrauterine growth retardation (IUGR). The third GBS positive mother delivered her baby at 25.2 weeks by primary C-section. The mother was admitted eight days prior to delivery and diagnosed with preterm premature rupture of membranes, and chorioamnionitis. She received ampicillin/sulbactam, Amoxicillin, and Erythromycin antepartum, and Cefazolin at the time the umbilical cord was clamped. The infant, in addition to being GBS positive with bacteremia, subsequently was diagnosed with Klebsiella pneumonia and expired 14 days after delivery. 

A total of 165 (71%) of the 231 mothers with unknown GBS status received antibiotic prophylaxis for GBS based on weak risk factor assessment. Of these 165 mothers, 106 (64%) delivered babies at <37 weeks gestational age, 1 (0.9%) had rupture of membrane with labor >18 hours, there were no mothers in this group with a temperature >38°C, and 105 (99%) had no recognized risk factors as outlined in the CDC guidelines [12] except for unknown GBS status. The flow diagram in Figure 1 shows the status of the women in the study.

## 4. Discussion

Our study demonstrates that in a private tertiary care hospital setting, both a high rate of screening for GBS and administration of intrapartum antibiotic prophylaxis can be achieved. No infants ≥37 weeks gestation developed invasive GBS disease. The three infants with early-onset GBS sepsis born to GBS positive mothers were less than 34 weeks gestation, and they would have been treated based on risk factors alone. The results for this study population are reflective of the overall population delivering at WHT.

A major strength of this study is our ability to capture data on GBS screening and usage of intrapartum antibiotic prophylaxis from our obstetrical database. Similar surveillance studies conducted by the CDC have been limited due to their inability to measure health care provider compliance with screening guidelines [14]. Despite our high rate of GBS screening, 11% of mothers had unknown GBS status at delivery. However, more than two-thirds of these women received prophylaxis for GBS based on risk factors alone. An additional strength is that the results reflect the management of GBS in a private tertiary care nonteaching obstetrical unit, with 80 practicing obstetricians, versus an academic hospital. The widely diverse nonhomogeneous patient population speaks to the nondiscriminatory nature of GBS colonization.

The retrospective nature of the study may be a weakness because the study sample was not controlled and was limited to a small proportion of the total deliveries at WHT. However, the study sample did represent 42% of all KSC deliveries during the two-year study period. 

Another weakness is that there were 11% (231) of mothers that were either not screened or data were not available. If a GBS positive rate of 29% is used, then this would contribute an additional 70 women to the GBS positive group. 

A confounding finding was that there were three newborns who developed early-onset GBS sepsis born to GBS positive mothers that were administered appropriate intrapartum antibiotic prophylaxis. It is not known whether or not GBS amnionitis was present at the time of admission to labor and delivery. None of these women had clinical signs or symptoms of infection. This emphasizes the fact that universal screening and intrapartum antibiotic prophylaxis cannot eliminate the occurrence of neonatal early-onset GBS sepsis.

Based on our experience reviewing GBS in mothers, we have undertaken an extensive review of neonatal outcomes in newborns of mothers receiving antibiotic prophylaxis within 4 hours of delivery. We also are developing a study to determine whether rapid screening using the PCR method in untested and GBS negative mothers will further reduce GBS infection in newborns.

## 5. Conclusion

This study found that adherence to the 2002 CDC guidelines for screening and prophylaxis for GBS could readily be accepted, and that in the first year of implementation, 89% of women delivering in a private tertiary care hospital with a large number of practicing obstetricians were screened. This retrospective study demonstrated that universal screening for GBS is effective and can be achieved in a private tertiary care hospital.

## Figures and Tables

**Figure 1:**